# Safe and efficacious artemisinin-based combination treatments for African pregnant women with malaria: a multicentre randomized control trial

**DOI:** 10.1186/1742-4755-12-5

**Published:** 2015-01-15

**Authors:** Michael Nambozi, Modest Mulenga, Tinto Halidou, Harry Tagbor, Victor Mwapasa, Linda Kalilani Phiri, Gertrude Kalanda, Innocent Valea, Maminata Traore, David Mwakazanga, Yves Claeys, Céline Schurmans, Maaike De Crop, Joris Menten, Raffaella Ravinetto, Kamala Thriemer, Jean-Pierre Van geertruyden, Theonest Mutabingwa, Umberto D’Alessandro, Pregact Group

**Affiliations:** Department of Clinical Sciences, Tropical Diseases Research Centre, Ndola, Zambia; Clinical Research Unit, Nanoro, Burkina Faso; Kwame Nkrumah University of Science and Technology, Kumasi, Ghana; College of Medicine, University of Malawi, Zomba, Malawi; Institute of Tropical Medicine, Antwerp, Belgium; Menzies School of Health Research, Darwin, Australia; International Health Unit, University of Antwerp, Antwerp, Belgium; Hubert Kairuki Memorial University, Dar es Salaam, Tanzania; Medical Research Council Unit, Serrekunda, The Gambia; London School of Hygiene and Tropical Medicine, London, UK; Pharmaceutical and Pharmacological Sciences Department, Leuven, KU Belgium

**Keywords:** Artemisinin-based therapy, Malaria in pregnancy, Pregnant women, Malaria, Sub-Saharan

## Abstract

**Background:**

Asymptomatic and symptomatic malaria during pregnancy has consequences for both mother and her offspring. Unfortunately, there is insufficient information on the safety and efficacy of most antimalarials in pregnancy. Indeed, clinical trials assessing antimalarial treatments systematically exclude pregnancy for fear of teratogenicity and embryotoxicity. The little available information originates from South East Asia while in sub-Saharan Africa such information is still limited and needs to be provided.

**Design:**

A Phase 3, non-inferiority, multicentre, randomized, open-label clinical trial on safety and efficacy of 4 ACT when administered during pregnancy was carried out in 4 African countries: Burkina Faso, Ghana, Malawi and Zambia. This is a four arm trial using a balanced incomplete block design. Pregnant women diagnosed with malaria are randomised to receive either amodiaquine-artesunate (AQ-AS), dihydroartemisinin-piperaquine (DHA-PQ), artemether-lumefantrine (AL), or mefloquine-artesunate (MQAS). They are actively followed up until day 63 post-treatment and then monthly until 4–6 weeks post-delivery. The offspring is visited at the time of the first birthday. The primary endpoint is treatment failure (PCR adjusted) at day 63 and safety profiles. Secondary endpoints included PCR unadjusted treatment failure up to day 63, gametocyte carriage, Hb changes, placenta malaria, mean birth weight and low birth weight. The primary statistical analysis will use the combined data from all 4 centres, with adjustment for any centre effects, using an additive model for the response rates. This will allow the assessment of all 6 possible pair-wise treatment comparisons using all available data.

**Discussion:**

The strength of this trial is the involvement of several African countries, increasing the generalisability of the results. In addition, it assesses most ACTs currently available, determining their relative ‘-value-’ compared to others. The balanced incomplete block design was chosen because using all 4-arms in each site would have increased complexity in terms of implementation. Excluding HIV-positive pregnant women on antiretroviral drugs may be seen as a limitation because of the possible interactions between antiretroviral and antimalarial treatments. Nevertheless, the results of this trial will provide the evidence base for the formulation of malaria treatment policy for pregnant women in sub-Saharan Africa.

**Trial registration:**

NCT00852423

## Background and rationale

The risk of malaria is higher in pregnant women than in the general population. There is insufficient information on the safety and efficacy of most antimalarial drugs in pregnancy [1] as they are systematically excluded from clinical trials for fear of teratogenicity and embryotoxicity [2]. This has complicated the generation of evidence-based recommendations for the prevention and treatment of malaria during pregnancy. Though the experience on the use of ACTs and their safety and efficacy in pregnancy is increasing (over 1,000 documented pregnancies, mainly in South East Asia), such information is still limited in sub-Saharan Africa [3]. Preclinical data indicate that the artemisinin derivatives were embryotoxic and potentially teratogenic in several animal species, without maternal toxic effects or impaired fertility [2]. More recent studies confirm these findings. One important aspect is that the critical window for drug exposure is approximately 10–14 days in the rat and their extrapolation to humans would indicate a sensitive period of weeks 2–6 of pregnancy [2]. There is increasing experience with the use of artemisinin derivatives in the second and third trimesters and there have been no reported adverse effects on the mother or foetus [4]. Despite limited data the World Health Organization (WHO) recommends effective artemisinin-based combination treatments (ACT) in the second and the third trimester and several African countries are already implementing it [3].

We propose to assess the efficacy and safety of the most important ACTs currently available, namely artemether-lumefantrine (AL), amodiaquine-artesunate (AQ-AS), mefloquine-artesunate (MQ-AS) and dihydroartemisinin-piperaquine (DHA-PQ). The choice of ACTs is based on several criteria, including known treatment efficacy in children, safety in pivotal phase-3 trials in children and adults, practicality of the dosing regimen (duration, e.g. 3 vs. 7 days), fixed dose combinations, drug tolerance, current availability in the population and affordable cost. The rationale for contemporaneously testing several drug regimens is to shorten the time of data collection and determine the relative ‘value’ of each treatment, providing the basis for an informed choice by malaria control programmes and policy makers.

Reliable data on the safety and efficacy of ACTs in pregnant women can be rapidly collected only within the context of a randomized-controlled trial. The production of such a large dataset will advance considerably the knowledge on the treatment of malaria in pregnancy in a relatively short period of time compared to pregnancy registers. One of the major issues for this trial is the altered pharmacokinetic of antimalarial drugs during pregnancy and the influence on the outcome of treatment. There has been a recent increase in trials that measure the pharmacokinetics of antimalarial drugs in pregnant women [5]. Recent information on the pharmacokinetics of both artemisinins [6] and partner drugs in the ACT is reassuring as amodiaquine [7], mefloquine [8] and piperaquine [9] do not seem to need dose adjustment. Nevertheless, the pharmacokinetic of lumefantrine (when administered as co-formulated artemether-lumefantrine) seems altered in pregnancy [10]. The clinical implications of the PK findings may not be clear from small rich PK studies alone and, unless the effects are important, may not be apparent in a pilot study in one site. Therefore, the proposed study aims not only at collecting safety and efficacy data on ACTs in a systematic and standardized manner but also explanatory variables (pharmacokinetic and *in vitro* drug sensitivity) that may help in interpreting the observed results.

ACTs have been shown to be extremely efficacious in children [11]. Efficacy is determined by the drug partnering the artemisinin derivative and, for artesunate–mefloquine, artemether–lumefantrine, and dihydroartemisinin–piperaquine, this usually exceeds 95% [12]. Amodiaquine-artesunate has proved to be an efficacious combination (range 90-95%) in areas where 28-day cure rates with amodiaquine monotherapy exceed 80% [13]. Few data on pregnant women are available but efficacy should be as good, if not better, than in children. In all the three areas where AQAS is assessed, efficacy of AQ monotherapy exceeds 80%. Therefore, it is expected that all the four treatments will have an efficacy of about 95%.

### Trial objectives and purpose

The main objective of the trial is to determine the safety and efficacy of 4 ACTs when administered to pregnant women with *P. falciparum* infection during the second and the third trimester, and collect explanatory variables for therapeutic response. The primary hypothesis is the clinical equivalence (pair-wise non-inferiority) of the 4 treatment regimens, with clinical equivalence defined as difference in treatment failure rates (PCR corrected) of 5% or less.

Specific objectives are:

- to compare the efficacy of AL, AQ-AS, MQ-AS and DHA-PQ in terms of treatment failure by 63 days after start of treatment with or without genotyping; Parasite clearance time; Haematological recovery by 14, 28, 42 and 63 days post-treatment and at delivery; Birth weight measured within 72 hours of delivery; and prevalence of placenta *P. falciparum* malaria;
- to describe the safety profile of AL, AQ-AS, MQ-AS and DHA-PQ in terms of tolerability; incidence of adverse events until one year post-partum;
- to determine the relation between drug pharmacokinetics (Day 7 levels of the partner drug) and response to treatment;
- to assess the *in vitro* susceptibility of *P. falciparum* isolates collected before treatment to several drugs, including the partner drug tested, and to correlate their IC_50_ to treatment response.

### Trial design

#### Study design

This is a non-inferiority, multicentre, randomized, open label study on 4 antimalarial treatments, namely DHA-PQ, MQAS, AQAS and AL, assessed in each site using a “balanced incomplete block design” – with 3 out of 4 arms used in each site. There are 7 sites in the study distributed in the four countries, i.e. Burkina Faso (Nanoro and Nazoanga), Ghana (Effiduase, Ejisu and Juaben), Malawi (Chikwawa) and Zambia (Nchelenge).

The treatments tested are distributed in a way to allow a head-to-head comparison and the establishment of the treatment’s relative value according to a series of outcomes (Table 1). This approach has the advantage of testing several treatment options at the same time, maximizing the use of resources, and is the most likely to achieve our aim of identifying at least 2 antimalarial treatments suitable for use in pregnancy and one rescue/alternative treatment.

**Table 1:** **Treatment arms per country/ site (number of patients)**

***West Africa: comparator AQAS***			
Burkina (870)	AL (290)	AQAS (290)	MQAS (290)
Ghana (870)	AQAS (290)	MQAS (290)	DHA-PQ (290)
***Eastern-Southern Africa: comparator AL***			
Malawi (870)	DHA-PQ (290)	AL (290)	AQAS (290)
Zambia (870)	MQAS (290)	DHA-PQ (290)	AL (290)

AQAS: amodiaquine-artesunate; DHA-PQ: dihydroartemisinin-piperaquine; AL: artemether-lumefantrine; MQAS: Mefloquine-artesunate.

The primary analysis is the assessment of therapeutic equivalence of the 4 treatments (clinical non-inferiority) with respect to therapeutic success at day 63 and their safety throughout the follow up, i.e. up to one year after delivery.

The following procedures are used to ensure an unbiased assignment of treatment safety and efficacy:

1. The randomization list is generated prior to the beginning of the study.
2. The interpretation of the PCR reading is blinded or masked with regard to the treatment allocation of the patients.
3. An independent Data Safety and Monitoring Board (DSMB) reviews all safety data.

#### Primary endpoint

There are two primary end points:

1. **Treatment Failure** (TF) (PCR adjusted) at day 63 defined according to the WHO criteria [14] as the sum of early and late treatment failures. Early Treatment Failure (ETF) could be one of the following: Development of danger signs or severe malaria on Day 0, Day 1, Day 2 or Day 3, in the presence of parasitaemia; Parasite density on Day 2 > Day 0 count, irrespective of axillary temperature; Presence of parasitaemia on Day 3 with fever (axillary temperature ≥ 37.5°C); Parasitaemia on Day 3 ≥ 25% of count on Day 0. Late treatment failure (LTF) is divided in late clinical and late parasitological failure. Late Clinical Failure (LCF): Development of danger signs or severe malaria on any day after Day 3 in the presence of parasitaemia, without previously meeting any of the criteria of Early Treatment Failure; Presence of parasitaemia and fever on any day after Day 3, without having previously meet the criteria of ETF. Late Parasitological Failure (LPF): Presence of parasitaemia on any day from day 7 onwards and axillary temperature <37.5°C, without previously meeting any of the criteria of ETF or LCF. The Adequate Clinical and Parasitological Response (ACPR) is 1-TF. It is defined as absence of parasitaemia at the end of the follow up period, irrespective of axillary temperature without previously meeting any of the criteria of early and late treatment failure. In the adjusted estimates, patients with late asexual parasite reappearance (with or without fever) are considered ACPR if the PCR analysis shows a new infection rather than a recrudescence.
2. **Safety profiles** including significant changes in relevant laboratory values. Subjects are monitored for 63 days for possible development of adverse events. All adverse events are recorded on the specific form in the electronic CRF. Vital signs, blood chemistry and haematology are monitored and changes in relevant laboratory parameters are assessed.

#### Secondary endpoints

PCR unadjusted treatment failure up to day 63 (TF63U); Time to treatment failure (PCR adjusted and unadjusted) during 63 days of active follow-up after treatment; Asexual parasite clearance time (PCT): Asexual parasite clearance time is defined as the time (in days) from time of randomization to 2 consecutive negative blood slides (collected at different days) - the time to the event is taken as the time to the first negative slide; Gametocytaemia (prevalence and density) at day 7, 14, 21, 28 and 63 after treatment; Hb changes day 14, 28, 42 and 63; Acute, chronic or past infection of the placenta (prevalence); Mean birth weight and prevalence of low birth weight.

#### Laboratory procedures

##### Haematology

Maternal haemoglobin is measured using Hb301 Hemocue*®*, Angelholm, Sweden, according to manufacturer’s instructions.

##### Peripheral malaria infection

Blood samples are collected by finger prick at specified time points during the trial for blood slides (thick blood film) and blood spots on filter paper. Thick blood smears are stained with 3% Giemsa for 30 minutes and read by trained microscopists at each site. Parasite densities are calculated by counting the number of asexual parasites per 200 leukocytes (or per 500 leukocytes if the count is <10 asexual parasites/200 leukocytes), assuming a leukocyte count of 8,000/μl. A blood smear is considered negative when the examination of 100 high power fields does not reveal asexual parasites. Each slide is read separately by two experienced microscopists and discrepancies resolved by a third reader.

Blood spots on filter paper are used for genotyping recurrent malaria infections during follow up and compared them with pre-treatment samples. This is done by characterizing MSP1, MSP2 and GLURP, single-copy genes in the *Plasmodium falciparum* genome. For the three genes, each PCR-amplification product of a different size is considered to originate from a different clone of *Plasmodium falciparum*, reflecting a different genotype. For the samples collected from the same patient at day 0 and day of recurrent parasitaemia, the length polymorphism of MSP1, MSP2 and GLURP are determined, i.e. the number of bands in each PCR reaction and their respective size. Results are interpreted as follows:

- Recrudescence: For each marker (MSP1, MSP2 and GLURP), at least one identical length polymorphism is found in the sample collected at day 0 and day of recurrent parasitaemia.
- New infection: For at least one marker, length polymorphism is different between the sample collected at day 0 and that at day of recurrent parasitaemia.
- Indeterminate: Samples that fail to produce a result due to an inability to amplify DNA at day 0 and/or day of recurrent parasitaemia.

##### Placental malaria

A 1 cm^3^ biopsy specimen is obtained from the maternal-facing side of the placenta as soon as possible after delivery. Biopsy specimens are preserved in 10% neutral buffered formalin which are processed and embedded in paraffin wax by standard techniques. Pending histological evaluation, all biopsies are kept at 4°C. Paraffin sections 4 mm thick are stained with haematoxylin-eosin stain.

Placental biopsies are classified according to the following definitions [15]:

1. Acute infection (parasite present, malaria pigment absent)
2. Chronic infection (parasites and malaria pigment present)
3. Past infection (no parasite but pigment present)
4. No infection (both parasites and malaria pigment absent)

##### PK assessment

Individual pharmacokinetic studies are often underpowered for identifying the factors that influence antimalarial pharmacokinetic parameters, which may have a major influence on the observed therapeutic response. Complementing efficacy data with some information on the pharmacokinetic properties of the treatment used allows a better interpretation of the observed recurrent infections or true recrudescences/new infections, as these may be the results of inadequate drug levels because of altered distribution, poor absorption or metabolism. The day 7 drug concentration has been shown to be the most important single measure, in terms of correlation with the area under the concentration time curve and association with treatment response, for lumefantrine, piperaquine and mefloquine [16]. Therefore, a blood sample of 2 ml is collected from all women at day 7 with the aim of measuring with an appropriate assay the concentration of the partner drug to the artemisinin derivative. Not all samples will be analysed; instead a smaller number of samples will be chosen for the analysis according to the observed therapeutic response, i.e. in each arm women having experienced a true recrudescence will be compared with those having had a new infection and with those with an adequate clinical and parasitological response. Analysis of the blood samples will be carried out within the Malaria in Pregnancy Consortium.

##### In-vitro tests

This component is carried out at the sites in Burkina Faso only. The sensitivity of the parasites to the drugs used is determined by carrying out *in vitro* tests. Venous blood samples (5 ml) are collected at day 0 before treatment from women with a parasite density of at least 4,000/μL. The HRP2 ELISA [17, 18] is used to measure the proliferation of *P. falciparum* in the presence of lumefantrine, monodesethylamodiaquine (active metabolite of amodiaquine), mefloquine, piperaquine and dihydroartemisinin (active metabolite of artemisinin derivatives).

#### Sample size

The sample size for this study was determined by simulation with the following assumptions and requirements: (1) study conclusions are determined by two-sided 95% confidence intervals for difference in response rates (% of therapeutic success), with decision rule as described below, (2) all 4 treatments have identical true response rates of 95%, (3) 95% power for each of the 6 pair-wise comparisons and 80% power for the combined hypothesis that all treatments are therapeutically equivalent is required. With these assumptions, approximately 700 patients/treatment arm are needed. If the true response rate for one of the treatments is lower than 95%, then power is reduced to 80% (for a true response rate of 94%) or 50% (for a true response rate of 93%). Allowing for a 20% loss to follow-up, a total of 870 patients are recruited to each treatment; this is equivalent to 290 patients in each treatment group in each centre (i.e. a total centre sample size of 870 patients) – and hence a total study sample size of 3480 patients. Inclusion of HIV-infected women is not expected to have a major influence on the sample size calculation. The percentage of therapeutic success may be slightly but not dramatically lower in this subgroup of patients.

For safety, when combined, the trial is able to detect with 90% power major adverse events occurring at the frequency of at least 2-3%.

#### Statistical analysis

A detailed analysis plan is drawn up prior to the analysis.

1. **Baseline comparability**: Patients in each treatment group in each site are described separately with respect to baseline characteristics. The clinical importance of any imbalance will be noted, though statistical tests of significance are not undertaken.
2. **Primary analyses**: The primary analysis of the study is the assessment of therapeutic equivalence of the 4 treatments (clinical non-inferiority) with respect to therapeutic success at day 63 and their safety throughout the follow-up, i.e. up to one year after delivery.

##### Efficacy

Therapeutic equivalence is assessed using the pair-wise difference in response rates (percentage of women with therapeutic success). Assessment of the difference in true response rates is performed by calculating the two-sided 95% confidence interval for the difference in response rates from the observed data, using the following decision rule:

- if the two-sided 95% confidence interval for the difference in response rates lies entirely between −5% and +5%, then therapeutic equivalence of the two treatments is concluded;
- if the 95% confidence interval for the difference in response rates includes −5% or +5%, then therapeutic equivalence cannot be established;
- if the 95% confidence interval for the difference in response rates lies entirely below −5% or entirely above 5%, then one treatment is clinically inferior to the other.

The primary analysis uses the combined data from all 4 centres together, with adjustment for any centre effects, using an additive model for the response rates (i.e. a generalized linear model with Bernoulli error distribution and an identity link function). This allows the assessment of all 6 possible pair-wise treatment comparisons using all available data. Equivalence will be established using two-sided confidence intervals. Though 6 treatment comparisons will be performed, no adjustment for multiplicity is needed as the focus of the study is on the individual pair-wise treatment comparisons. In addition, combined hypotheses of interest (e.g. all 4 treatments are therapeutically equivalent) require each of the individual hypotheses to be accepted. Consequently, there is no inflation of the type I error rate due to multiple testing. However, the power for combined hypotheses is lower than for the individual pair-wise comparisons. Thus, the power calculation of this study required a high (95%) power for individual pair-wise treatment comparisons, resulting in an acceptable (80%) power for the combined hypothesis. For the efficacy analysis, both an intention-to-treat and a per-protocol approach are adopted, with the per-protocol analysis being the primary approach, as recommended for equivalence studies.

##### Safety

For safety analysis, all non-serious and serious adverse events (SAE) are grouped according to a pre-specified side-effect coding system and tabulated. The number (and percentage) of patients experiencing any adverse event, any SAE, and any drug-related SAE are compared between treatment groups using Fisher's exact test. Safety is analyzed using the all-patients-treated approach.

#### Selection of the patients

All pregnant women in the second and third trimester (<37 weeks) and attending the antenatal clinic of the study health facilities are systematically screened for malaria infection with a rapid diagnostic test; if positive they are further assessed for eligibility. They are included if they are at least 15 years old, with a pregnancy of at least 16 weeks, a *P. falciparum* monoinfection of any density, regardless of symptoms, and a Hb concentration of at least 7 g/dL. Pregnant women with a negative blood slide are not included in the study and go through the routine antenatal clinic procedures according to national policy and receive a dose of sulfadoxine-pyrimethamine (SP) as intermittent preventive treatment (IPT). Exclusion criteria include history of allergic reactions to the study drugs, of known pregnancy complications or bad obstetric history such as repeated stillbirths or eclampsia, of presence or history of major illnesses likely to influence pregnancy outcome, e.g. diabetes mellitus, severe renal or heart disease, or active tuberculosis, current cotrimoxazole prophylaxis or ARV treatment. Table 2 provides the full list of inclusion/exclusion criteria. The reason of including women with any parasite density is justified by the important adverse outcomes any malaria infection has on the mother’s and her offspring’s heath. Limiting the inclusion to women at 16 weeks or more of gestation is justified by the uncertainty on the safety of ACT when administered during the first trimester of pregnancy. The gestational age was confirmed by measuring symphysio-fundal height and the foetal viability by using an ultra-sonography. Exclusion criteria are formulated because of possible safety issues, e.g. history of allergic reactions to the study drugs, or the need for a clear interpretation of the therapeutic response, e.g. recent exposure to antimalarial treatments.

**Table 2:** **Inclusion and exclusion criteria**

Inclusion criteria	Patients eligible for inclusion in the trial must fulfil all of the following criteria
	Gestation ≥16 weeks and <37 weeks;
	*P. falciparum* monoinfection of any density, with or without symptoms
	Hb ≥ 7 g/dL;
	At least 15 years old;
	Residence within the health facility catchment’s area;
	Willing to deliver at the health facility;
	Willing to adhere to the study requirements (including, in Zambia and Malawi, HIV VCT)
	Ability to provide written informed consent; if the woman is minor of age/not emancipated, the consent must be given by a parent or legal guardian according to national law (however, in this case, the investigator is responsible to check that the woman herself is also freely willing to take part in the study).
**Exclusion criteria**	**Patients who meet any of the following criteria are not eligible for the study**
	History of allergic reactions to the study drugs;
	History of known pregnancy complications or bad obstetric history such as repeated stillbirths or eclampsia;
	History or presence of major illnesses likely to influence pregnancy outcome including diabetes mellitus, severe renal or heart disease, or active tuberculosis;
	Current cotrimoxazole prophylaxis or ARV treatment;
	Any significant illness at the time of screening that requires hospitalization, including severe malaria;
	Intent to move out of the study catchment area before delivery or deliver at relative’s home out of the catchment area.
	Prior enrollment in the study or concurrent enrollment in another study.
	Unable to take oral medication
	Clear evidence of recent (1 week) treatment with antimalarials or antimicrobials with antimalarial activity (clindamycin; azythromycin; etc.)

##### Informed consent

For the informed consent, all interviews are conducted in the native language of the patients by a qualified person identified by the Investigator. Written information and consent forms in the local language are provided to the women or Legally Authorized Representatives (LAR) for their review. After the interview, the patients and, in case they are of minor age/not emancipated, the parents or guardians are asked to confirm their willingness to participate in the study by signing (or thumb-printing if illiterate) the consent form.

Each eligible pregnant woman who agrees to give informed consent is assigned a unique study number and enrolled. Besides malaria infection and parasite density, Hb, total white blood cell count, differential count, total bilirubin, ALAT and creatinine are measured. In addition, a blood sample is collected on filter paper for later genotyping.

#### Randomization and treatment

Randomisation is carried out according to a pre-established list comprising blocks of varying size and stratified according to the number of recruitment points in each site. Allocation of treatment according to the randomization list is in sealed envelopes labelled with the patient’s unique code, guaranteeing concealment until recruitment**.**

The study treatment is administered during the first 3 study days (days 0–2) by the study doctor or nurse and the patient kept for one hour in the clinic. If vomiting occurs within 30 minutes, a full treatment is re-administered, half a dose if after 30 minutes. In case of persisting vomiting, an alternative treatment, e.g. quinine, is provided.

#### Patients follow up

Scheduled visits are at day 3, 7 and then every week until day 63 post-treatment. However, women are encouraged to attend the antenatal clinic if they felt ill between scheduled visits. At the end of the active follow-up period, women are asked to attend the antenatal clinic monthly and at any time they feel unhealthy until delivery. At each visit, scheduled and unscheduled, the medical history since the last visit (including any treatment taken), current signs and symptoms (if any) are collected. A blood sample for thick and thin blood film and later genotyping to determine the rate of re-infection is collected and the body temperature checked. Haematology (Hb, total white blood cell count, differential count) is measured at day 7, 14, 28 and 63; biochemistry (total bilirubin, ALAT and creatinine) at day 7 and 14.

If pregnant women recruited during the third trimester deliver before the end of the 63-day active follow, scheduled visits continue after delivery until the day 63 is completed. The outcome of pregnancy, including any congenital abnormality, the birth weight and maternal Hb are collected as soon as possible after delivery. In addition, a placenta impression smear and a placenta biopsy for later histopathological analysis are collected. Both the mother and the new-born are reassessed twice after delivery for any adverse event: between 4 and 6 weeks and then after one year (Table 3). Antimalarials or antibiotics with antimalarial activity (erythromycin or other macrolides, co-trimoxazole or other sulphonamides, any tetracycline including doxycycline, and quinolones, clindamycin) cannot be administered during the active follow up as it would lead to withdrawal of the patient from the study.

**Table 3:** **Study procedures/study visit schedule**

Day	0	1	2	3	7	14	21	28	35	42	49	56	63	Any other day ^1^	Delivery	4-6 weeks post-partum	EPI clinics	1 year post-partum
History (symptoms)	X																	
Informed consent	X																	
Examination (clinical)	X	X	X	X	X	X	X	X	X	X	X	X	X	X	X			
Foetal viability	X			X	X	X	X	X	X	X	X	X	X	X				
Blood Pressure	X	X	X	X	X	X	X	X	X	X	X	X	X	X	X			
Temperature	X	X	X	X	X	X	X	X	X	X	X	X	X	X	X			
Blood film	X	X	X	X	X	X	X	X	X	X	X	X	X	X	X			
Filter paper PCR	X				X	X	X	X	X	X	X	X	X	X	*X* ^2^			
Adverse events	X	X	X	X	X	X	X	X	X	X	X	X	X	X	X			
Concomitant medications	X	X	X	X	X	X	X	X	X	X	X	X	X	X	X	X		
Hb	X				X	X		X		X			X		X			
Haematology	X				X	X		X					X		X			
Treatment	X	X	X															
Blood sample for PK					X													
Pop PK	3 samples/woman according to a predefined schedule (120 women in 3 sites each)																	
*In vitro* test	X	and time of recurrent infection																
Biochemistry	X				X	X		X^3^					X^4^					
Placenta sample															X			
New born assessment															X	X		
Infant assessment																	X	X

^1^Spontaneous attendance to the health facility.

^2^Includes placental blood sample.

^3^Only ALAT and total bilirubin.

^4^Only ALAT and total bilirubin, and only if abnormal at Day 28.

Patients with treatment failure, including parasitological failure, are treated with rescue treatment (quinine 10 mg/kg orally three times a day for 7 days or an anti-malarial treatment according to the country’s national guidelines) and their active follow up stopped. Nevertheless, they are still followed up (safety data) until one year post-delivery.

#### Safety variables

Safety is closely monitored during the course of the study in compliance with ICH/GCP guidelines. At each visit, the investigator ascertains the occurrence of any adverse events since the previous visit; including those involving laboratory values which are out of normal range and are of clinical importance to be considered as AEs, and the proper AE reporting procedure is followed. The severity of a clinical adverse event is scored according to the following scale: mild, moderate, severe and life-threatening.

Serious Adverse Events (SAEs) are defined as any untoward medical occurrence that, at any dose of the medication given, fulfilled the following criteria; death, life-threatening, requiring hospitalization or prolongation of hospitalization, resulting in persistent or significant disability or incapacity, congenital anomaly/ birth defect, or other situations such as important medical events that may not be immediately life-threatening or result in death or hospitalization but may jeopardize the subject or may require medical or surgical intervention to prevent one of the other outcomes listed in the above definition. The reporting investigator assesses the relationship between investigational product and the occurrence of each AE/SAE. The relationship of an adverse event to study drug is assessed according to the following definitions: ‘Definitely unrelated’, ‘Unlikely’, ‘Possible’, ‘Probable’ and ‘Definitely related’. The outcome of each AE is assessed according to the classification as follows – ‘Completely recovered’, ‘Not yet completely recovered’, ‘Deterioration’, ‘Permanent damage’, ‘Death’, ‘Ongoing’ and ‘Unknown’.

Each SAE has to be reported to the sponsor and to the concerned ethical bodies in the study countries within 24 working hours since the time the study staff becomes aware of it, and any reporting delay has to be explained. All the SAE forms are further sent by the sponsor to the concerned ethical bodies in Belgium and to the independent DSMB. Each SAE is followed up until resolution.

#### Monitoring and quality assurance

Each site is visited at least 3 times during the conduct of the trial plus a study initiation visit at the start of clinical activities and a close-out visit after the last patient has completed the follow up. The monitor will perform the tasks as described in International Conference on Harmonisation (ICH)-Good Clinical Practice (GCP) E6, section 5.18 and will carry out at least 10% source data verification (SDV). For all sites, the SDV percentage will be increased by the monitor if the quality of data entry is found not to be satisfactory.

#### Case report form and data management

Each patient has her own source document file, according to a common source document template provided by the Sponsor, with all the original documents, e.g. laboratory results. This data is captured into an electronic case report form (e-CRF) developed in the GCP-compliant software MACRO (InferMed, UK) for clinical trials. The e-CRF has in-built consistency checks; data can be entered either online or offline and then uploaded to be sent to the central server. The final database is obtained after the resolution of all queries and then locked for later statistical analysis done according to a pre-established statistical data analysis plan.

#### Study committee

##### Consortium secretariat

The Consortium Secretariat (CS) acts as a steering committee. It comprises one investigator from each site and will assess the progress of the trial. The members of the CS will address policy and operational issues related to the protocol. The CS has responsibility for protecting the scientific conduct and integrity of the trial. Its functions include review of the protocol before ethical approval; and formulation of recommendation for any change in the design and operations of the trial during the course of the trial, when needed.

##### Data safety and monitoring board

The independent Data Safety and Monitoring Board (DSMB) is composed by four independent scientists, i.e. a paediatrician, a gynaecologist, a statistician and a malariologist. They meet every three months during recruitment or can be called together if the necessity arises.

##### History of amendments to the study protocol

Several amendments (Table 4) have been made to this study protocol based on emerging information and were approved by the relevant ethical committees. Before study start the ASAQ manufacturer provided information regarding potential transitory increase of ALAT at day 28 post-treatment. Therefore, ALAT measurement at day 28 was introduced. In addition, an overview of the reproductive toxicity of DHAPQ and mefloquine was made available by the manufacturer and showed in the animal model prolonged length of gestation and dystocic pup expulsion in animals treated close to delivery. It was therefore, decided to modified the original inclusion criterion of gestation ≥16 weeks to gestation ≥16 weeks and <37 weeks.

**Table 4:** **Major amendments**

Amendment version	Basis/rationale of amendment	Amendment
Amendment 1	The information provided by the ASAQ manufacturer of some transitory increase of ALAT at day 28 post-treatment.	ALAT measurement at day 28 was introduced
Amendment 2	The reproductive toxicity of DHAPQ and mefloquine was made available by the manufacturer and showed in the animal model prolonged length of gestation and dystocic pup expulsion in animals treated close to delivery.	Modification of the original inclusion criterion of gestation ≥16 weeks to gestation ≥16 weeks and <37 weeks
Amendment 3		paragraph on placenta biopsy is added to the ICF (was omitted by mistake in the previous versions)
Amendment 4	Amendment was done on the basis of the modification of blood piperaquine concentrations by food intake which could result in a QTc prolongation, a risk factor for serious cardiac arrhythmia.	• DHA/PQP tablets should be administered with water only and at least 3 hours apart from meal, mainly for the second and third administration.
		• correction of dosage of DHA/PQP, should be 3 tablets for 3 consecutive days instead of 2 tablets for 3 consecutive days as erroneously stated in the previous versions of the protocol.
		• notification of change in sites in Malawi.
		• % of SDV reduced to 10%
Amendment 5	Need for baseline drug plasma concentrations	PK sample at day 0 before study drug administration for the patients participating in the population PK study
Amendment 6		one additional site

Another amendment was done on the basis of the modification of blood piperaquine concentrations by food intake which could result in a QTc prolongation, a risk factor for serious cardiac arrhythmia. Therefore, the manufacturer advised to administer DHAPQ tablets with water only and at least 3 hours apart from meal, mainly for the second and third administration, and advice the woman not to eat for the next 3 hours.

## Discussion

Pregnant women are one of the high risk groups affected by the malaria burden and few antimalarials are available to treat them. This study assesses the efficacy and safety of four ACTs for the treatment of uncomplicated malaria in pregnant women in Africa. This is the largest clinical trial of its kind and will provide the evidence base for the formulation of treatment guidelines for malaria in pregnancy.

Evidence of the interaction between malaria and HIV-1 has already been reported. HIV-1 infected pregnant women have a higher prevalence of peripheral parasitaemia and placental malaria [19, 20] and their infants experience higher postnatal mortality when both diseases are present [4, 21]. HIV-1 infected adults have a higher risk of malaria infection and clinical malaria, the latter increasing with falling CD4-cell count [22, 23]. Therefore, offering adequate and efficacious antimalarial treatment and prevention is extremely important for this high risk group. However, little is known about the safety and efficacy of antimalarial drugs in HIV-infected individuals and much less on the interaction between antimalarials and antiretrovirals [24] and reliable data are urgently needed [3]. HIV-infected individuals have a higher risk of experiencing treatment failure and this depends on the degree of immune-suppression [25]. However, no major safety problems related to ACT treatment in pregnant women have been identified so far.

Considering that most pregnant women recruited in the study will have an infection with a relatively low parasite density and that they will be treated with an ACT, it is expected that the number of treatment failures in this specific sub-group would be extremely low. They will have to be treated in any case as the consequences of the malaria infection on the woman’s health and that of her offspring are well known.

In the second and third trimester of pregnancy, WHO recommends the use of ACT known to be effective in the country/region. Despite this recommendation, it should be recognised that the available information on the treatments to be used in this trial is limited. ACT should not be used in the first trimester of pregnancy, the time of greatest concern for potential teratogenicity, and particular care will be taken in excluding women of this gestational age. DHA-PQ is the least used of the 4 ACTs studied in this trial and it is not among the WHO recommended ACTs during pregnancy because of insufficient information [3]. However, DHA-PQ is the first line treatment in Papua Indonesia, where it is also used to treat pregnant women with malaria; an observational study reported significant benefits of DHA-PQ over quinine-based regimens in reducing recurrent malaria and poor foetal outcome in pregnant women in the second or third trimester [26]. This trial will increase significantly the knowledge on the use of DHA-PQ in African pregnant women.

## Trial status

Data collection completed.

## Abbreviations

ACPR: Adequate clinical and parasitological response
ACT: Artemisinin combination therapy
AE: Adverse event
AL: Artemether lumefantrine
ALAT: Alanine amino-transferase
AQAS: Amodiaquine artesunate
ARV: Antiretroviral
CRF: Case report form
CS: Consortium secretariat
DHA-PQ: Dihydroartemisinin piperaquine
DNA: Deoxynucleic acid
DSMB: Data safety and monitoring board
ELISA: Enzyme-linked immunosorbent assay
ETF: Early treatment failure
GCP: Good clinical practice
GLURP: Glutamate-rich protein
HIV: Human immunodeficiency virus
HRP: Histidine-rich protein
ICH: International conference on harmonisation
IPT: Intermittent preventive therapy
ITN: Insecticide treated net
LAR: Legally authorised representative
LCF: Late clinical failure
LPF: Late clinical failure
LTF: Late treatment failure
MIP: Malaria in pregnancy
MQAS: Mefloquine artesunate
MSP: Malaria merozoite surface protein
PCR: Polymerase chain reaction
PCT: Parasite clearance time
PK: Pharmacokinetics
SAE: Serious adverse event
SDV: Source data verification
SP: Sulfadoxine pyrimethamine
TF: Treatment failure
VCT: Voluntary counselling and testing
WHO: World health organisation.
